# Building genetic tools in *Drosophila* research: an interview with Gerald Rubin

**DOI:** 10.1242/dmm.025080

**Published:** 2016-04-01

**Affiliations:** Gerry Rubin is a Howard Hughes Medical Institute (HHMI) Vice President and the Executive Director of the Janelia Research Campus, as well as John D. MacArthur Professor of Genetics, Emeritus, at the University of California, Berkeley. Gerry is a member of the National Academy of Sciences, the Institute of Medicine, the American Academy of Arts and Sciences, and a foreign member of the Royal Society (UK).

## Abstract

Gerald (Gerry) Rubin, pioneer in *Drosophila* genetics, is Founding Director of the HHMI-funded Janelia Research Campus. In this interview, Gerry recounts key events and collaborations that have shaped his unique approach to scientific exploration, decision-making, management and mentorship – an approach that forms the cornerstone of the model adopted at Janelia to tackle problems in interdisciplinary biomedical research. Gerry describes his remarkable journey from newcomer to internationally renowned leader in the fly field, highlighting his contributions to the tools and resources that have helped establish *Drosophila* as an important model in translational research. Describing himself as a ‘tool builder’, his current focus is on developing approaches for in-depth study of the fly nervous system, in order to understand key principles in neurobiology. Gerry was interviewed by Ross Cagan, Senior Editor of Disease Models & Mechanisms.

## Abstract (precis)

***Drosophila* Collection:** Ross Cagan spoke to Gerry Rubin about his career path and the story behind some of his key breakthroughs, as well as his unique and successful approach to management and mentorship.


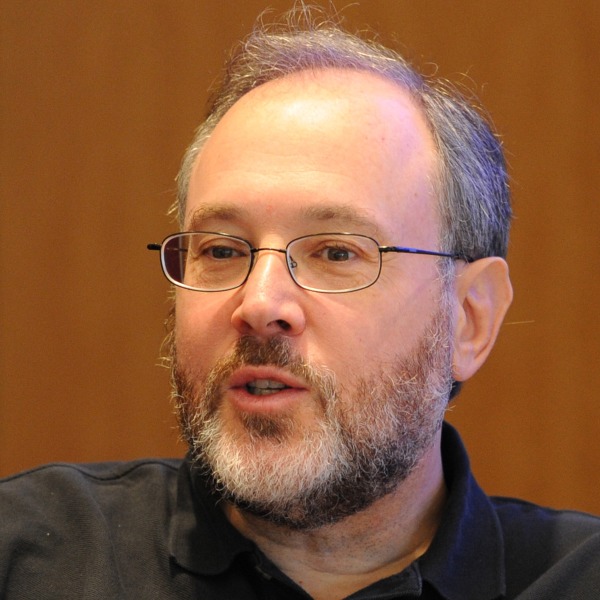


Gerald M. Rubin was born in Boston, Massachusetts, in 1950. He undertook his undergraduate degree in Biology at MIT from 1967 to 1971, and worked at Cold Spring Harbor Laboratory during the summers of 1970 and 1971. For his PhD, he studied 5.8S ribosomal RNA under the supervision of Sydney Brenner at the MRC Laboratory of Molecular Biology, University of Cambridge. After graduating in 1974, Gerry undertook post-doctoral research in David Hogness's lab at Stanford University. This marked Gerry's first step into *Drosophila* research, which at that time was experiencing resurgence in the biomedical arena. Gerry's first faculty appointment was at Harvard Medical School, but he moved after 4 years to the Carnegie Institute of Washington's Department of Embryology. It was here that, together with Allan Spradling, Gerry pioneered the use of transposable elements as a tool for genetic manipulation – a breakthrough that revolutionized *Drosophila* genetics. In 1983, Gerry moved to the University of California, Berkeley, where his group studied pattern formation and signal transduction during development of the fly eye; he later led the sequencing of the fruit fly genome, which was completed in 2000. Currently, Gerry is a Vice President at Howard Hughes Medical Institute and the Executive Director of its biomedical research institute, the Janelia Research Campus in Ashburn, Virginia. His lab probes fly brains to gain insight into the neuronal circuits underlying learning and memory, sleep regulation, visual perception and sensory integration, and Gerry remains dedicated to the development of neurobiology and genomics tools. He has been honored with a long list of awards in recognition of his contributions to *Drosophila* research so far.

**My first question is: how did you become interested in flies?**

I was a graduate student in Cambridge, England, in the pre-cloning, pre-DNA-sequencing era, working on RNA sequencing in yeast. David Hogness came and gave a seminar in 1973 on his recent work on fly chromosomes. At that time, many labs, including his, were trying to develop methods for cloning segments of eukaryotic DNA in bacteria. Dave's work really fascinated me and prompted me to apply to do my post-doctoral research in his lab. He agreed, and I was ready to move to Stanford in September 1974. By then, recombinant DNA technology was up and running, and the Hogness lab had generated about 200 *Drosophila* recombinant clones. My first project was to make a library that had enough clones in it to cover all the DNA sequences in the genome. This was the first of several times in my career when I was fortunate to be in the right place at the right time. There had been almost no prior work on cloned DNA segments, so almost any experiment led to novel insights. The experience also taught me the power of new techniques to advance science.

**You and Allan Spradling pioneered the use of transposable P elements as a gene manipulation tool – this is one of the first things I learned about in *Drosophila* genetics. How did you come to be involved in this work?**

Most of my post-doc time was spent characterizing the organization of fly DNA by making restriction maps of clones, measuring the amount of repetitive DNA (using reassociation kinetics – readers over the age of 50 will remember this technique!) and performing *in situ* hybridization to polytene chromosomes. We found a lot of repetitive DNA dispersed in the fly genome. Around the same time, Eric Davidson at Caltech had proposed that repetitive DNA segments might have important regulatory roles, so I continued studying them after setting up my own lab at Harvard. We soon realized that many of them were retroviral transposable elements and that they were causing spontaneous mutations in the genome. There was a growing body of literature on movable genetic elements, and Mel Green – among others – had proposed that their insertion caused some of the unstable mutations that had been described in *Drosophila*. We decided to try to clone the *white* locus – a gene that is important for eye color in *Drosophila* – because the many spontaneous mutations at this locus would allow us to directly test these ideas. In 1980, we succeeded in cloning the locus, by a method now known as transposon tagging, and we were soon able to show that several mutations in the *white* locus were in fact due to transposon insertion. That same year, I went to the Cold Spring Harbor symposium on transposable elements and heard Bill Engels give a talk on hybrid dysgenesis, which he proposed was caused by a family of transposons called P elements. In the slow lunch line following Engel's talk, Margaret Kidwell, Paul Bingham and I designed an experiment to generate and molecularly characterize hybrid-dysgenesis-induced mutations in *white*. If Engel's hypothesis were correct, the mutant *white* genes would contain insertions of the postulated P element. This turned out to be the case.

At Carnegie, I went on to characterize the structure of a large number of P elements. We discovered that there were full-length elements with a conserved structure, and a heterogeneous set of smaller elements containing internal deletions. This was immediately reminiscent of Barbara McClintock's pioneering work in maize that defined ‘two-element systems’ of autonomously transposing and defective elements dependent on the autonomous elements for *trans*-acting functions. At that point it was obvious that the full-length P elements might be good vectors for making transgenic animals. Allan Spradling, my colleague at Carnegie, and I discussed trying to make this work. Allan took on the task of injecting embryos and I did most of the molecular work. It took us less than a year to have initial success and we reported our work in two articles in *Science* in the fall of 1982. We did all the experiments ourselves because it was a high-risk ‘nutty’ experiment!

**The breakthrough dovetailed nicely with the burst of genetic screens that were happening around the same time. Did you know, when you were doing this work with Allan, the impact it would have?**

We realized, from the work in yeast and bacteria, that the ability to make transgenic animals would be tremendously powerful. Methods to genetically engineer the genomes of single-cell organisms had been around for a while, but we were the first to successfully engineer the germline of a multicellular organism. A lot of people had been interested in doing this, and we were fortunate that the experiments we tried actually worked. A lot of things fell into place and it was remarkably efficient and successful. It was one of the few times you have in your career as a scientist when everything falls into place and the sort of dream experiment actually works the way you draw it up on paper. Once we had the system up and running, we passed on the reagents and so, by the time our paper was published, several other labs had already confirmed that it worked.

The P-element-based approach came along at a good time because everyone was hungry for positional cloning. Everyone had in mind the genes they wanted to identify and the experiments they wanted to do. There were a lot of fascinating developmental mutants, for example those isolated in the Nobel-Prize-winning genetic screens of Nüsslein-Volhard and Wieschaus, that hadn't been characterized on a molecular level – we had no idea how the mutations were affecting cellular processes. The field then really took off and I think it was the synergy between molecular cloning and all the mutants and techniques that we already had that meant we could finally get at molecular mechanisms. A tremendous amount of data came out very quickly from many laboratories, particularly on developmental processes, and it was information that we hadn't been able to glean using classical embryology in flies – or indeed any other organism. The majority of the components of all the signal transduction pathways that exist in humans were first discovered through this work. We could now ask questions that were just not possible to ask beforehand. It was as though the whole field awakened because of the sudden leap in several key technologies.

**Your decision to share the reagents was critical. What made you decide to do this?**

There is a tradition to share among *Drosophila* researchers, going back to the days of Thomas Morgan. The fly field has always seemed much more open in this respect than the mammalian field. Allan and I considered our options: we had this very useful technique and could wait until it was published to distribute it – which would give us exclusive access for 6-8 months – or to give it to people straight away. We decided that the benefits of distributing it immediately would outweigh any progress we could make in our work within a few months. I learned a lot from the experience and think it one of the better decisions I have made in my life. I consider myself to be a tool builder – the thing I'm most proud of in my scientific career is the development of tools and methods. Post-docs in my lab were eager to test hypotheses, but what has always motivated me is the building of tools that help overcome technical obstacles that block progress.

“I consider myself to be a tool builder – the thing I'm most proud of in my scientific career is the development of tools and methods”

**You led the fly genome project that was completed back in 2000. How did you get involved with that and what did the experience teach you?**

My lab was doing interesting work on eye development at the time, and I took on the task of leading the genome project with some reluctance. Allan and I felt that the fly genome really needed to be sequenced, and we realized that we should take the lead because we were well-established and in the position to be doing something for the community. We got a grant and recruited some younger scientists, and I became the ‘figurehead’ of the project. Over time many of the younger scientists got bored or were hired by industry, and I needed to spend more and more of my time on the project. But I had staked my reputation that the project would be completed. So I was glad to be able to collaborate with Celera Genomics and Craig Venter, which was not only very enjoyable and a tremendous help in getting the project completed, but also demonstrated the power of the whole-genome shotgun method for sequencing large genomes. Once the fly sequence was available, I happily got out of the genome sequencing field and returned to the interesting questions that could now be asked about gene organization and function.

One important thing my genomics experience taught me was that I like management. I'm one of the few scientists that I know who actually enjoys management and thinks it is, in its own way, as interesting and challenging as scientific discovery and research. Managing projects, managing people, getting people to work well together and doing collaborative interdisciplinary research projects is a unique kind of challenge. This combination of enjoying scientific discovery as well as management naturally led me to my current role at Janelia.

**Let's talk a little bit about Janelia, which you have seen evolve from concept to implementation. What was the thinking behind its approach to interdisciplinary research, and what do you hope the long-term impact of the campus will be?**

I feel that the way research is done in the US nowadays, where scientists depend for their livelihood on getting their grants renewed, can have a profound negative effect on research. It can make the work short-sighted by forcing scientists to pursue directions depending on what the funding agencies want, rather than by following their instincts and passions. I have always felt that this isn't the ideal way to do all science. I moved to Janelia at a time when collaborative research and tool building were also undervalued and underappreciated. If someone wanted to build a new optical microscope, physics departments wouldn't hire them because optics is old physics, and they wanted string theorists. In biology, people cared only about the hypothesis being tested and not about the tools. The tools are needed to advance science, yet science wasn't – and still isn't – funded to align well with this. There is a lack of appreciation for basic research in general, although basic science brings the fundamental breakthroughs that underlie translational research. Just look at CRISPR/Cas9, which would never have emerged if we didn't have people studying ‘weird’ phenomena in bacteria. The pressure for recipients of grants to show short-term return on investment by tackling ‘practical’ problems – often defined top-down by funding agencies – is something I have seen steadily increase during my 40 years in science.

“There is a lack of appreciation for basic research in general, although basic science brings the fundamental breakthroughs that underlie translational research”

I felt that places like the MRC Laboratory of Molecular Biology [LMB], where I was fortunate enough to do my PhD, had a much higher rate of innovative research. I began to think about what made such places so great. As they were internally funded, people didn't worry about convincing grant review committees to fund their research. Decisions got made much more quickly, the labs were small and people didn't have other responsibilities; they could keep working in the lab, even at a late stage in their career. At the MRC LMB, I would see Nobel Prize winners with a pipette in hand, doing experiments 8 hours a day, 5 days a week. You would never see that in the US, and I had a rude awakening when I came back here.

I wanted to create a supportive environment for aspiring scientists who really wanted to just keep doing science without having to manage an enterprise and raise funds. And where there would be a strong synergy between the work in individual labs. I think Janelia provides that kind of environment. It's not right for everyone – maybe it's only right for 5% of scientists – but for those people it provides something very special that would be difficult to find elsewhere. My short-term goal is to help all the talented people we have recruited to be successful in doing important, innovative science that would be unlikely to happen elsewhere. My ultimate goal is to have a disruptive impact on scientific culture as a whole, and to change the way a significant fraction of research is funded and scientists are evaluated, especially in the US, by demonstrating the success of an alternative model. I look at Janelia as an experiment in the sociology of science and we continue to refine our working hypothesis. But I think the initial data from our first 10 years are very encouraging.

“My ultimate goal is to have a disruptive impact on scientific culture as a whole, and to change the way a significant fraction of research is funded and scientists are evaluated, especially in the US, by demonstrating the success of an alternative model”

**What would you say to a young scientist thinking about entering the fly field, particularly in terms of the place of *Drosophila* in this brave new world of translational research?**

I think there's no denying that *Drosophila* has been very useful as a disease model. Many components of signal transduction pathways, which are important drug targets, were discovered in yeast, worms or flies. People are working on very interesting experiments relating to growth control, cell movement and other basic mechanisms that are important in disease contexts. Drawing from my own scientific interests, I think there is a lot that flies can contribute to neurobiology. Flies show complex behavior, and I believe that the basic rules about how a biologically constructed computational device can perceive the world, navigate through it, learn and remember things, can be learned by studying flies. Flies are particularly amenable to a ‘black box approach’ in science: studying the consequences of manipulating genes and cells using clever assays can yield information that doesn't depend on having a good hypothesis. You can gather information, in a non-biased way, on how things work.

“Flies are particularly amenable to a ‘black box approach’ in science: studying the consequences of manipulating genes and cells using clever assays can yield information that doesn't depend on having a good hypothesis”

From an intellectual point of view, I think that working on an organism like *Drosophila* is highly rewarding. Certainly for students, the ability to design and do really interesting experiments and see the outcome in a reasonable timeframe is tremendously powerful in terms of learning how to do science. In other fields, experiments can move so slowly that if you're a graduate student you don't really get to do much during the limited time you have for your PhD. The difficulty lies in convincing the funding agencies of the value of model systems such as *Drosophila*. There is insufficient appreciation from people making these decisions, most of who have worked in medicine or using mammalian systems, about the power of a simpler model. The work going on in vertebrate systems is important, but I think that the simpler systems deserve more investment than they get at present. Dollar for dollar the fly community has produced more insights with medical relevance than the mouse community. Similarly, in the cancer field, most of the important cell cycle mutants and checkpoints initially came from studies in yeast. The intellectual history, the source of important ideas and breakthroughs, isn't always fully appreciated, and thus the contribution of simple systems is undervalued.

**You've mentioned in the past that one of your biggest achievements in science is the people you have trained. Certainly, as somebody on the outside looking in, you've had an astonishing number of amazing post-docs that have gone on to make an impact. What is your approach to training?**

It's true that this is one of the things I'm most proud of. It certainly is as rewarding as a good publication record, and of course it's a two-way street. The fact that I had all those great people in my lab made it easy for me to develop my own reputation based on the work they did.

I was fortunate as a graduate student; I pretty much worked on my own. I never wanted anyone telling me what to do. I was the sole author of two of the three papers I published as a graduate student; on the other one I had a co-author but it wasn't my adviser. I had my own project and it moved in the direction I chose. I think most people are more motivated when they're working on their idea and not an idea that someone else gives them. I was also fortunate because I entered a field that was in its early stages. The *Drosophila* community was growing and there weren't that many labs who had established research programs using recombinant DNA and so I attracted to my lab a remarkable group of highly talented people. I realized that the way I would get the most out of them was to let them work on a problem they were passionate about, tell them they could take that problem with them when they left my lab, and just be on hand to provide technical advice and to act as a sounding board for ideas or help with writing manuscripts. Smart people want to be in an environment where they have freedom to explore their ideas and are challenged by other smart people. It was wonderful for me because every day there was interesting science going on, and I never felt like I needed to be the source of all the ideas or that everything was on my shoulders. I was creating an environment that facilitated other people's ability to work, rather than directing them. As a young scientist, I was always in an environment that was positive, upbeat and optimistic. I feel tremendously fortunate and feel that I have a big obligation to try and recreate some of that for the next generation.

**What advice would you give to a young start-up on how to run a successful lab?**

Keep it small and be highly selective. This advice seems counterintuitive: you're thinking, you have an empty lab, that you have some money to hire people and any reasonable pair of hands is better than nothing. But actually, if you get the wrong person, it can be disastrous. Aim to hire people smarter or more talented than yourself. Having people who are not motivated and passionate can be very detrimental – motivation and passion are probably as important as intellectual ability. Also, you should protect your time so that you can continue to do things yourself at the bench.

“Having people who are not motivated and passionate can be very detrimental – motivation and passion are probably as important as intellectual ability”

**Finally, if not science, what would you be doing?**

I got my first job working in a lab when I was 14 years old (washing glassware) and I knew right from that point that I wanted to work in science. I suppose if I hadn't gotten a faculty job I would be teaching high-school biology. As I've mentioned, I've been extremely fortunate in every stage of my career. I've been in the right place at the right time on several occasions and I got tremendous support from my advisers and teachers – as well as from my wife of 38 years. My graduate and post-doc experiences were also extremely positive. I can't imagine anything else I could have done that would have been as satisfying or as much fun – I never looked at it as work. I'm always amazed that I get paid for doing what I love doing.

